# Competitive binding-based optical DNA mapping for fast identification of bacteria - multi-ligand transfer matrix theory and experimental applications on *Escherichia coli*

**DOI:** 10.1093/nar/gku556

**Published:** 2014-07-10

**Authors:** Adam N. Nilsson, Gustav Emilsson, Lena K. Nyberg, Charleston Noble, Liselott Svensson Stadler, Joachim Fritzsche, Edward R. B. Moore, Jonas O. Tegenfeldt, Tobias Ambjörnsson, Fredrik Westerlund

**Affiliations:** 1 Department of Astronomy and Theoretical Physics, Lund University, Sölvegatan 14A, 223 62 Lund, Sweden; 2 Division of Chemistry and Biochemistry, Department of Chemical and Biological Engineering, Chalmers University of Technology, Kemivägen 10, 412 96 Göteborg, Sweden; 3 Division of Solid State Physics, Department of Physics, Lund University, PO 118, 221 00 Lund, Sweden; 4 Department of Infectious Diseases, Sahlgrenska Academy, University of Gothenburg, Guldhedsgatan 10A, 413 46 Göteborg, Sweden; 5 Department of Applied Physics, Chalmers University of Technology, Kemivägen 10, 412 96 Göteborg, Sweden

## Abstract

We demonstrate a single DNA molecule optical mapping assay able to resolve a specific *Escherichia coli* strain from other strains. The assay is based on competitive binding of the fluorescent dye YOYO-1 and the AT-specific antibiotic netropsin. The optical map is visualized by stretching the DNA molecules in nanofluidic channels. We optimize the experimental conditions to obtain reproducible barcodes containing as much information as possible. We implement a multi-ligand transfer matrix method for calculating theoretical barcodes from known DNA sequences. Our method extends previous theoretical approaches for competitive binding of two types of ligands to many types of ligands and introduces a recursive approach that allows long barcodes to be calculated with standard computer floating point formats. The identification of a specific *E*. *coli* strain (CCUG 10979) is based on mapping of 50–160 kilobasepair experimental DNA fragments onto the theoretical genome using the developed theory. Our identification protocol introduces two theoretical constructs: a *P*-value for a best experiment-theory match and an information score threshold. The developed methods provide a novel optical mapping toolbox for identification of bacterial species and strains. The protocol does not require cultivation of bacteria or DNA amplification, which allows for ultra-fast identification of bacterial pathogens.

## INTRODUCTION

Base-by-base genome sequencing is continuously becoming faster and less expensive but issues still exist that have not been solved. An important limitation is the short read lengths (<1 kilobasepairs (kb)) that cause long-range information to be lost (1,2). Optical mapping was pioneered in the 1990s by Schwartz *et al.* (3) as a complement to base-by-base sequencing. Based on the use of restriction enzymes that cut DNA stretched on a surface, the lengths and positions of the fragments formed were analyzed, using fluorescence microscopy, to create a ‘barcode’ of the analyzed DNA. Since then, several different strategies for optical mapping, with improved resolution, have been developed (4). Optical maps allow visualization of coarse sequence information on mega-base-pair DNA fragments and have found use in a variety of different applications ranging from genome assembly (5) to the detection of structural gene variations (6) and the identification and characterization of microorganisms (7).

In recent years, nanofluidic channels have been extensively used for the optical mapping of stretched DNA molecules (8). Several groups have applied specific labeling schemes to create optical maps. Jo *et al.* (9) demonstrated an enzymatic approach to tag specific sequences and similar approaches have been reported by Das *et al.* (10), Lam *et al.* (11) and Neely *et al.* (12).

To avoid the use of enzymatic reactions and tailored substrates, Reisner *et al.* (13) created a sequence-specific fluorescence pattern along individual stretched DNA molecules, by partial denaturation of the DNA inside nanochannels. Using a combination of formamide and heat denaturation they generated local melting of stretched DNA stained with the fluorescent dye, YOYO-1 (YOYO). Since AT-rich regions have a lower free energy of dissociation than GC-rich regions, they denature at a lower temperature and the dye will dissociate preferably from these regions. The result is a fluorescence pattern along the DNA molecule that reflects the underlying sequence, with a resolution of ∼1 kb. Welch *et al.* (14) later used the assay to map single DNA pieces extracted from a gel plug (<300 kb) onto the genome of *Saccharomyces cerevisiae*. The same principle was used by Marie *et al.* to study structural variations on mega-base-pair-long DNA fragments extracted from human metaphase chromosomes (15).

**Figure 1.**
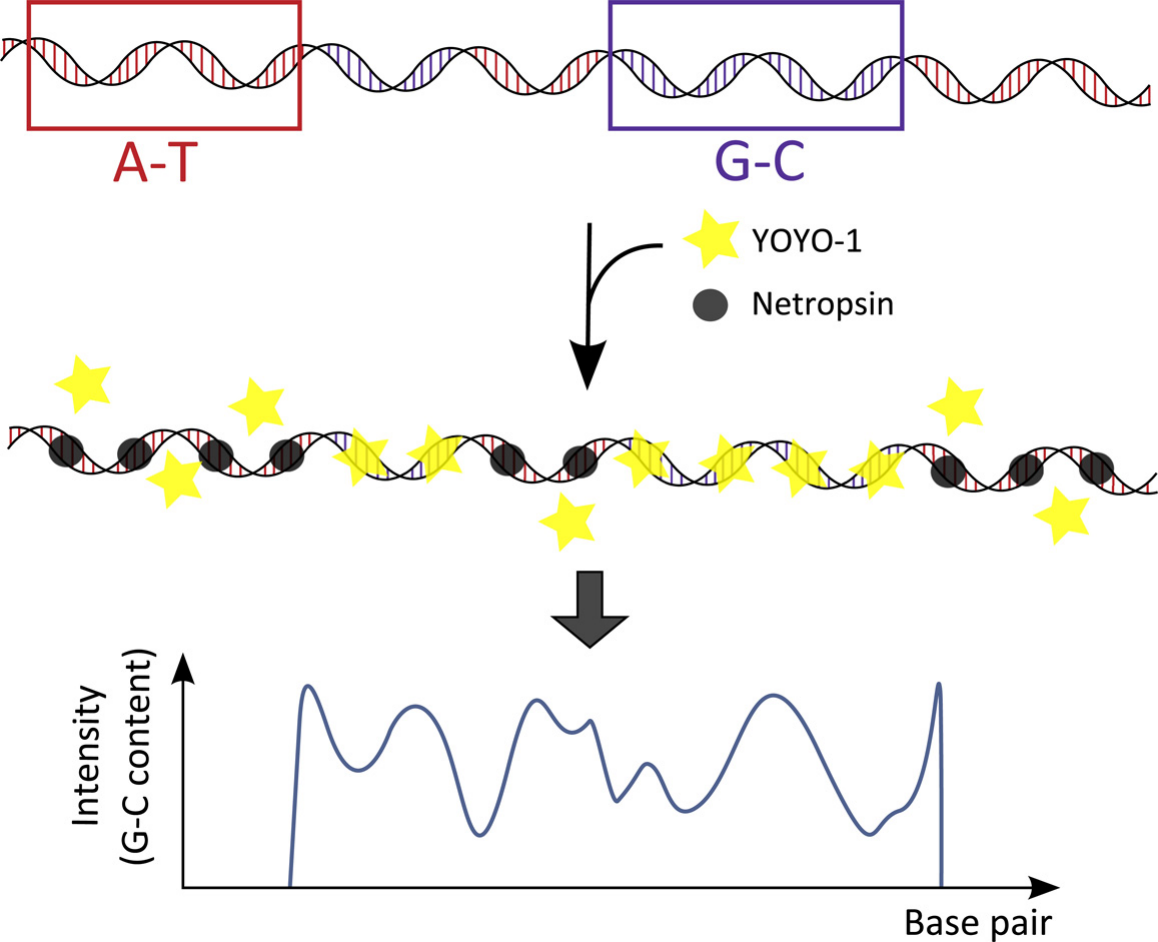
Schematic illustration of the principle of the CB assay. YOYO-1 (yellow stars) and netropsin (gray circles) are simultaneously added to a DNA with AT- rich (red) and GC-rich (blue) regions. Netropsin binds preferentially to AT-rich regions preventing YOYO-1 to bind to these regions. When stretched in nanofluidic channels the DNA molecules show an emission intensity along the contour that reflects the underlying sequence with bright GC-rich and dark AT-rich regions.

As an alternative to DNA melting, we have recently demonstrated how competitive binding (CB) between YOYO and the natural antibiotic netropsin can be used to create optical maps of single, nanoconfined DNA molecules (16). Netropsin binds in the minor groove of DNA and has a very strong preference for binding to AT-rich sequences (17,18). When DNA is added to a mixture of YOYO and netropsin, the two molecules will compete for the AT-rich binding sites and the result is an emission intensity along the DNA contour that reflects the underlying sequence, where GC-rich regions appear bright and AT-rich regions dark (Figure 1). Proof-of-principle CB experiments were performed on commercially available DNA from lambda and T4 phages, demonstrating that the emission patterns observed reflect the underlying sequences in a predictable way (16).

To further strengthen the CB barcoding technology, it is crucial to make theoretical statistical physics predictions to relate experimental intensity patterns to DNA sequences. The problem of CB of ligands covering more than one lattice site (base-pair) on a one-dimensional lattice (here, DNA molecule) has a long history (19,20). These earlier studies assumed one ligand type and binding constants were taken to be independent of site (base-pair composition). For this scenario, an analytic expression for the average occupancy of sites as a function of ligand concentration was derived—the McGhee-von Hippel binding isotherm—using probabilistic arguments. In (21) the same binding isotherm was derived using a site-independent transfer matrix approach. More recent interest in this problem (22–24) stems from the applicability of transcription factor binding to DNA (25–28). To include also site dependence into the CB problem, one has to resort to numerical schemes such as the site-dependent transfer matrix approach (27,28).

In this study, we extend the CB optical mapping technique in several ways: (i) we optimize the conditions to obtain reproducible barcodes with as much information as possible by mixing the samples at high ionic strength to speed up equilibration and subsequent dilution to low ionic strengths (29); (ii) we extend the transfer matrix method (21,27,28) from being applicable to two types of ligands, to site-dependent multi-ligand CB; (iii) to circumvent numerical problems associated with multiplication of many (∼10^6^) transfer matrices, we introduce a novel recursive approach that allows long barcodes to be calculated with standard computer floating point formats; (iv) to compare theoretical predictions to experimental data, we match experiments for 50–160 kb DNA fragments extracted from an *Escherichia coli* strain (CCUG 10979 = ATCC 8739) to the corresponding theoretical barcode; (v) we demonstrate that it is possible to identify a specific strain of *E. coli* from a reference database of nine *E. coli* genome sequences. Our identification protocol consists of two theoretical constructs: a *P*-value for a best experiment-theory match and an information score (IS) threshold. The developed methods provide a promising and novel protocol for using optical CB maps for the identification of bacterial species and strains. We expect the methods to find applications in, for example, clinical diagnostics.

## MATERIALS AND METHODS

### Experiments

A stock solution containing YOYO-1 (Invitrogen), netropsin (Sigma-Aldrich) and DNA was prepared in 5× TBE buffer (Medicago, 10× TBE tablets) to the desired concentration and was then wrapped in foil and allowed to set for 10 min at room temperature. To prepare the loading sample, the stock solution was carefully diluted (1:100) to 0.05× TBE and 4% β-mercaptoethanol (v/v) was added to suppress photo-nicking of the DNA. The resulting DNA concentration in the loading sample was 0.5 μM (bp), the YOYO concentration was 0.1 μM and the netropsin concentration was 15 μM. T4GT7 DNA was obtained from Nippon Gene and purchased through Wako. The length of the *E. coli* DNA fragments was approximated using the length of lambda-DNA (48.5 kb, New England Biolabs) stretched in channels with the same dimensions as reference.

*E. coli* strain CCUG 10979 (synonymous with ATCC 8739, Acc. No. NC_010468.1) was cultured on blood agar medium (5% defibrinated horse blood; Substrate Department, Sahlgrenska University Hospital, Sweden) at 30 °C, and then re-cultured at the same conditions, over night. Biomass from two plates was suspended in 3 ml EDTA-saline (NaCl 0.15M, EDTA 0.01M, pH 8). The bacterial suspension was incubated with lysozyme at 37 °C for 30 min. Sodium Dodecyl Sulphate (SDS) was added to the suspension that was then vortexed and incubated at 65 °C for 10 min. NaCl was added and the sample was vortexed. Chloroform: isoamylalcohol (24:1) was added to the sample, shaken for 20 min and centrifuged (17 900 g, 15 min). The upper phase was collected and the chloroform extraction repeated. The upper phase was again collected and AcNa and isopropanol was added, precipitating the DNA. The precipitated DNA was collected by rolling on a closed Pasteur pipette, and then dissolved in water and further purified by incubation with RNase for 2 h at 37 °C and Proteinase K for 1 h at 37 °C. The purification (chloroform) and precipitation (isopropanol) was performed again, as above. The resulting DNA was stored at −20 °C.

The nanofluidic chips were fabricated in fused silica, using conventional techniques, as described in detail elsewhere (8). One chip holds two separate compartments, where each compartment consists of four wells that are connected in pairs via micro channels that in turn are connected by an array of nanochannels. The nanochannels have the following dimensions: ∼100 nm × 150 nm or ∼100 × 100 nm (height × width) and a length of ∼500 μm. The loading sample was applied to the chip, using a syringe, and transferred to the nanochannel array by pressure-driven flow. To make the DNA molecules enter the nanochannels, pressure was applied over two connected microchannels simultaneously. All the data was recorded, using a Zeiss AxioObserver.Z1 microscope equipped with a 100× TIRF oil immersion objective (NA = 1.46) from Zeiss and a Photometrics Evolve EMCCD camera. Image stacks of 100 images were recorded for each molecule using an exposure time of 200 ms.

To obtain a time-averaged experimental ‘barcode’, one must account for center-of-mass diffusion in the channel and conformational fluctuations. To that end, we applied a slightly modified version of the ‘local box stretching’ approach in (30). We complemented the algorithm by applying a moving average on the experimental signal, for alignment purposes. We also introduced a rough method for aligning the start and finish pixel of the region containing the DNA (see Supplementary Information for details and for an analysis of the noise properties of the aligned experimental barcodes). See Figure 4 for an example of the result of the alignment and data fitting.

### Quantifying the quality of experimental barcodes

We here define two quantities, the signal-to-background ratio (SBR) and Information Score (IS), which we use for characterizing experimental barcodes.

The SBR is defined as

(1) \begin{equation*} {\rm SBR} = \frac{\langle {\rm DNA \ signal}\rangle -\langle {\rm background}\rangle }{\langle {\rm background}\rangle } \end{equation*}

where ‘DNA signal’ refers to the signal in the region containing the DNA molecule and background is the signal outside of this region. Both numerator and denominator above denote averages over their respective regions.

The IS associated with a DNA barcode quantifies the quality and sharpness of a barcode, for a given microscope setup (31). IS is defined as

(2) \begin{eqnarray*} &&{\rm IS}= -\sum _k \log \nonumber \\ &&\left(\frac{1}{\sqrt{2\pi \log (\sigma ^2+\chi )}} \exp \left(- \frac{(\log (|\Delta I(k)|)^2}{2\log (\sigma ^2+\chi )}\right)\right) \end{eqnarray*}

where Δ*I*(*k*) is the difference between two neighboring ‘robust’ (see below) peaks and valleys in the barcode. The parameter *χ* is a regularization parameter, introduced to make sure that IS remains positive and real; we choose *χ* = 1. The background variance is denoted by σ^2^, see (31) for details on how σ^2^ is computed. In the same article a computationally efficient method for identifying robust peaks and valleys, i.e. regions which to the left and right are surrounded by barriers larger than some threshold *I*_threshold_, was described. Figure S1 in the Supplementary Information displays an example of a barcode with its robust extrema marked. Throughout this study we use *I*_threshold_ = σ.

### The multi-ligand transfer matrix method

Consider the theoretical problem at hand: *S* ligand species, labeled by *s* (*s* = 1, 2, ..., *S*), competing for binding to a DNA lattice with *N* base pairs (see also Figure S3 in the Supplementary Information). The ligands have bulk concentrations, *c*_*s*_, covering λ_*s*_ base pairs when bound to the DNA, and have site-dependent binding constants, *K*_*s*_(*i*), where *i* corresponds to the base-pair location along the DNA. For later purposes, we need to differentiate between different parts of the ligands; to that end, a ligand of type *s* is said to be composed of λ_*s*_ ‘monomers’. In the experiments performed and described herein, we have two types of ligands (*S* = 2), netropsin and YOYO, both of which occupy four base pairs when bound to DNA (λ_1_ = λ_2_ = 4). Without loss of generality, the binding constants, *K*_*s*_(*i*), are assigned to the left-most site occupied when the respective ligands bind (27). Cooperativity is included through cooperativity parameters, $\sigma _{\it {\it s},s^{\prime }}$, that add the possibility to include cooperative interactions between the two competing ligands, (*s* ≠ *s*′), as well as between the ligands themselves (*s* = *s*′).

**Figure 2.**
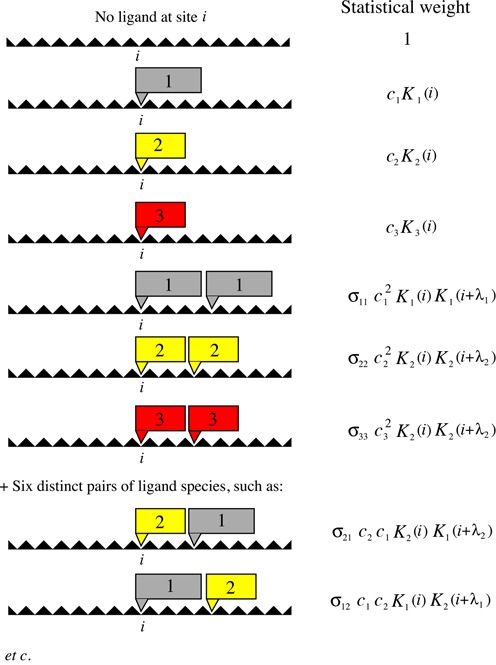
List of the different statistical weights for a base-pair *i* when in contact with bulk consisting of *S* different ligand species (here, *S* = 3 for illustrative purposes). The quantity *c*_*s*_ (*s* = 1, ..., *S*) is the bulk concentration of ligand type *s*, *K*_*s*_ is the associated binding constant and $\sigma _{s,s^{\prime }}$$\sigma _{s,s^{\prime }}$ are the different cooperativity parameters between the ligands species.

The goal of the theoretical calculations is to calculate the probability, *p*_*s*_(*i*), that a base-pair *i* is occupied by (one of the monomers of) a ligand of type *s*. To that end, we here introduce an extension of the transfer matrix approach, described in (27,28) for two types of ligands, to multi-ligand CB. As in (27,28) we write:

(3) \begin{equation*} p_s(i) = \frac{Z_s(i)}{Z} \end{equation*}

where *Z* is the partition function and *Z*_*s*_(*i*) is a sum over all allowed Boltzmann-weighted states consistent with base-pair *i* being covered by a type *s* ligand. Below we show that *Z*_*s*_(*i*) and *Z* can be calculated using transfer matrices. The various statistical weights needed for the transfer matrix approach are illustrated in Figure 2.

To proceed, we need to enumerate all possible states for a given base-pair *i*. We choose to use *m* as a label for the different states and employ an enumeration scheme as follows (see also Figure S4 in the Supplementary Information): state *m* = 1 corresponds to site *i* being unoccupied; states 2 to λ_1_ + 1 are states wherein the site is occupied by different monomers of type *s* = 1 (‘gray’) ligand; states λ_1_ + 2 to λ_1_ + λ_2_ + 1 correspond to states wherein the sites are occupied by different monomers of type *s* = 2 (‘yellow’) ligand, etc. There are, in total, $M=\sum _{\alpha =1}^S \lambda _\alpha +1$ number of states for each base pair.

**Figure 3.**
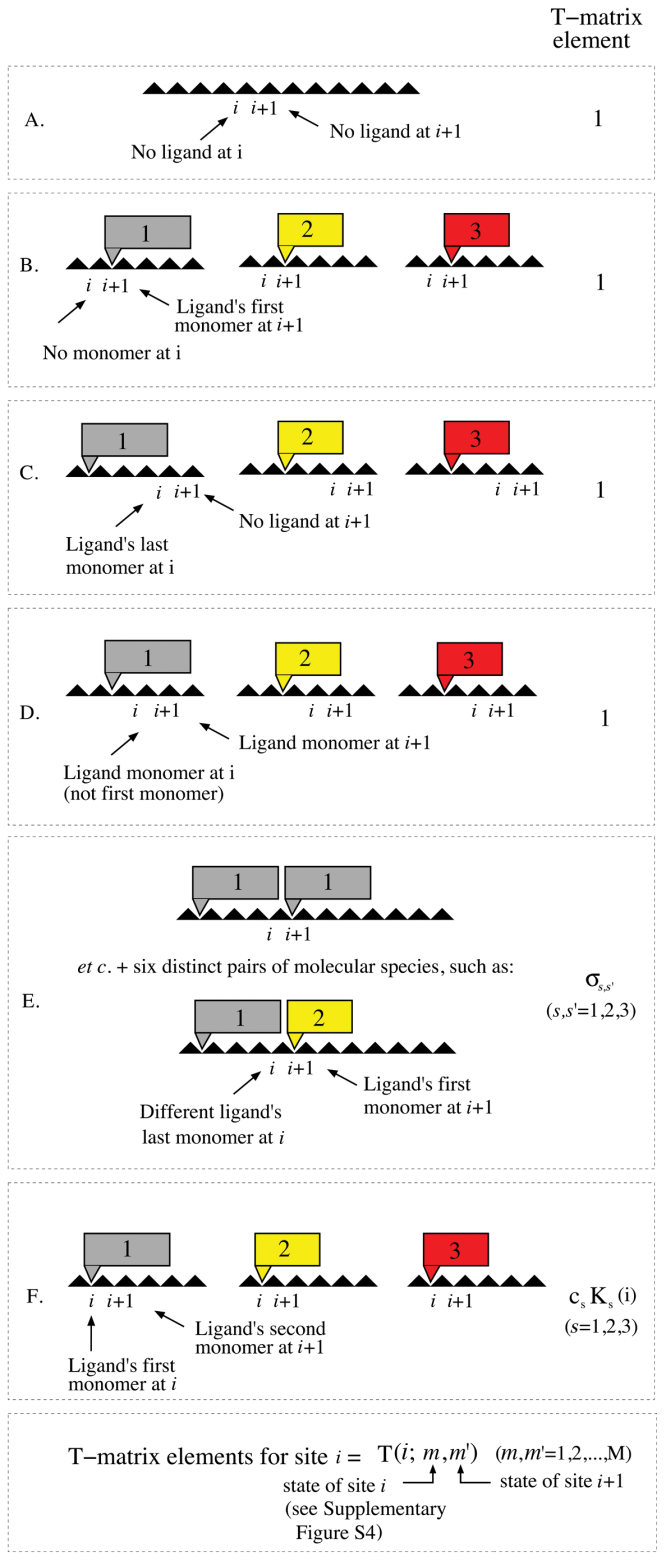
Explicit transfer matrix elements for site *i* in a multi-ligand setting. Conditioned that site *i* + 1 is in one of its allowed states (see Figure S4 in the Supplementary Information) site *i* can be in one of the states listed. Associated with each such pair of states (at sites *i* + 1 and site *i*) is a transfer matrix element value as given in the figure, and further detailed in Equations (2)–(7) in the Supplementary Information. These results are valid for arbitrary numbers of ligand types, even though we in this figure limit ourselves to three types of ligands (*S* = 3), for illustrative purposes.

We are now in a position to introduce the transfer matrices (27,28). Briefly, for each base pair we introduce an *M* × *M* transfer matrix ${\boldsymbol{T}}(i)$ with elements *T*(*i*; *m*, *m*′). These matrix elements give the statistical weight for site *i* to be in state *m* provided that site *i* + 1 is in state *m*′. Most of the elements in the transfer matrix are zero since, for example, if site *i* + 1 is occupied by the last monomer of a type 1 ligand, then site *i* cannot also be occupied by the last monomer of another type 1 ligand (if λ_1_ ≥ 2). With the statistical weights presented in Figure 2 and our choice of enumeration in mind, it is straightforward to provide expressions for the elements of the transfer matrix ${\boldsymbol{T}}(i)$. Explicit results are given in Figure 3. In the Supplementary Information, we provide explicit forms for *T*(*i*:*m*, *m*′) which allow straightforward automated computation of these transfer matrices for arbitrary *S* and λ_*i*_ (see Equations (2)–(7) in the Supplementary Information).

For the experiments presented in the Results section, we have two ligand species that cover four base pairs when bound, i.e. λ_1_ = λ_2_ = 4. For this case, each site has a total of *M* = 9 possible states and the associated 9 × 9 transfer matrices are:

\begin{equation*} {\boldsymbol{T}}(i) = \left( \begin{array}{ccccccccc}1 & 0 & 0 & 0 & 1 & 0 & 0 & 0 & 1 \\ 1 & 0 & 0 & 0 & \sigma _{1,1} & 0 & 0 & 0 & \sigma _{1,2} \\ 0 & 1 & 0 & 0 & 0 & 0 & 0 & 0 & 0 \\ 0 & 0 & 1 & 0 & 0 & 0 & 0 & 0 & 0 \\ 0 & 0 & 0 & c_1K_1(i) & 0 & 0 & 0 & 0 & 0 \\ 1 & 0 & 0 & 0 & \sigma _{2,1} & 0 & 0 & 0 & \sigma _{2,2} \\ 0 & 0 & 0 & 0 & 0 & 1 & 0 & 0 & 0 \\ 0 & 0 & 0 & 0 & 0 & 0 & 1 & 0 & 0 \\ 0 & 0 & 0 & 0 & 0 & 0 & 0 & c_2K_2(i) & 0 \\ \end{array} \right) \end{equation*}

Note that there are only *S* (here, *S* = 2) elements that are site-dependent in the general case, see case F in Figure 3 and Equation (7) in the Supplementary Information.

The partition function *Z* [see Equation (3)] is now (27,28)

(4) \begin{equation*} Z = {\boldsymbol{v}}(1)^T \cdot {\boldsymbol{T}}(1) \cdot {\boldsymbol{T}}(2) \cdot \cdot {\boldsymbol{T}}(N) \cdot {\boldsymbol{v}}(N+1) \end{equation*}

wherein two column vectors of length *M*, ${\boldsymbol{v}}(1)$ and ${\boldsymbol{v}}(N+1)$, are introduced: ${\boldsymbol{v}}(1)$'s elements are zero except for $v$(1; 1) = 1 and $v(1;1+\sum _{\alpha =1}^{s} \lambda _{\alpha }) = 1$ (for all *s* = 1, 2, ..., *S*). Thus, ${\boldsymbol{v}}(1)$ contains a list of all allowed states for base-pair 1. Similarly, we introduce a vector ${\boldsymbol{v}}(N+1)$ with all zero elements, except for $v$(*N* + 1; 1) = 1, guaranteeing that base-pair *N* can only be in its allowed states, namely unoccupied or covered by the last monomer of one of the *S* ligand species (27). We do not allow for ligand ‘overhang’ at the ends of the lattice, i.e. a ligand must have all its monomers attached to the lattice (DNA molecule) for binding to be allowed. For CB to ultra-long DNA molecules at optical (kb) resolution, as considered in the Results section, end effects due to different boundary conditions are negligible.

The number of Boltzmann-weighted configurations, *Z*_*s*_(*i*) [Equation (3)], constrained so that site *i* is occupied by a ligand of type *s*, can also be calculated, using transfer matrices. We have

(5) \begin{eqnarray*} &&Z_s(i) = \nonumber \\ &&{\boldsymbol{v}}(1)^T \cdot {\boldsymbol{T}}(1) \cdot \cdot {\boldsymbol{T}}(i-1) \cdot {\boldsymbol{O}}_s \cdot {\boldsymbol{T}}(i) \cdot \cdot {\boldsymbol{T}}(N) \cdot {\boldsymbol{v}}(N+1) \end{eqnarray*}

where we introduce projection operator ${\boldsymbol{O}}_s$, which projects onto states wherein one of the monomers of ligand *s* is bound to site *i*. Explicitly, these matrices have elements = 0, except for elements *O*_*s*_(*m*, *m*) for $m\in \lbrace 2+\sum _{\alpha =1}^{s-1}, \lambda _\alpha , ..., 1+\sum _{\alpha =1}^{s} \lambda _\alpha \rbrace$ which are = 1. For instance, multiplication by the matrix ${\boldsymbol{O}}_1$ onto ${\boldsymbol{T}}(i) \cdot {\boldsymbol{T}}(i+1) \cdot \cdot \cdot {\boldsymbol{T}}(N) \cdot {\boldsymbol{v}}(N+1)$ certifies that only states 2 to λ_1_ + 1 (see Figure S4 in the Supplementary Information), i.e. states where bp *i* is covered by one of the monomers of a type 1 ligand, are retained. For the case of interest in the Results section (λ_1_ = λ_2_ = 4), we have explicit projection operators:

\begin{equation*} {\boldsymbol{O}}_1 = \left( \begin{array}{ccccccccc}0 & 0 & 0 & 0 & 0 & 0 & 0 & 0 & 0 \\ 0 & 1 & 0 & 0 & 0 & 0 & 0 & 0 & 0 \\ 0 & 0 & 1 & 0 & 0 & 0 & 0 & 0 & 0 \\ 0 & 0 & 0 & 1 & 0 & 0 & 0 & 0 & 0 \\ 0 & 0 & 0 & 0 & 1 & 0 & 0 & 0 & 0 \\ 0 & 0 & 0 & 0 & 0 & 0 & 0 & 0 & 0 \\ 0 & 0 & 0 & 0 & 0 & 0 & 0 & 0 & 0 \\ 0 & 0 & 0 & 0 & 0 & 0 & 0 & 0 & 0 \\ 0 & 0 & 0 & 0 & 0 & 0 & 0 & 0 & 0 \\ \end{array} \right) \end{equation*}

and, similarly for ${\boldsymbol{O}}_2$, which has zero elements, except for *O_2_*(6, 6) = *O_2_*(7, 7) = *O_2_*(8, 8) = *O_2_*(9, 9) = 1.

### Stabilized transfer matrix calculations for long DNA

Direct numerical implementation of Equations (3), (4) and (5) together with the explicit transfer matrices above allows us to compute theoretical CB profiles for *S* ligand species for small lattices (typically *N* < 1000). However, a direct implementation for large *N*, as done in (27), is not numerically feasible, since matrix multiplications then ‘explode’ exponentially, causing numerical floating point precision problems. Below we show how to remedy this problem.

Let us now describe our stabilized recursive transfer matrix method. The computational time of the method scales linearly with the number of base-pair *N*. We utilize four sets of vectors and two sets of numbers, according to (*i* = 1, 2..., *N*)

(6) \begin{eqnarray*} {\boldsymbol{u}}^L(i) = {\boldsymbol{w}}^L(i-1) \cdot {\boldsymbol{T}}(i) & \qquad {\boldsymbol{u}}^R(i) = {\boldsymbol{T}}(i) \cdot {\boldsymbol{w}}^R(i+1) \nonumber \\ n^L(i) = |{\boldsymbol{u}}^L(i)| & \qquad n^R(i) = |{\boldsymbol{u}}^R(i)| \nonumber \\ {\boldsymbol{w}}^L(i) = \frac{{\boldsymbol{u}}^L(i)}{n^L(i)} & \qquad {\boldsymbol{w}}^R(i) = \frac{{\boldsymbol{u}}^R(i)}{n^R(i)} \end{eqnarray*}

The set of equations above constitutes recursion relations, which can be evaluated numerically, using ‘initial’ conditions, ${\boldsymbol{w}}^L(0)={\boldsymbol{v}}^T(1)/|{\boldsymbol{v}}^T(1)|$ and ${\boldsymbol{w}}^R(N+1)={\boldsymbol{v}}(N+1)/|{\boldsymbol{v}}(N+1)|$; this procedure requires *N* matrix multiplications. Note that vectors with index *L* (‘left’) are row vectors, whereas vectors with an index *R* (‘right’) are column vectors. Numerical stability is gained by normalizing the vectors ${\boldsymbol{u}}^L(i)$ and ${\boldsymbol{u}}^R(i)$, using normalization constants *n*^*L*^(*i*) and *n*^*R*^(*i*), respectively, after each matrix multiplication. This normalization procedure is a main contribution of this study and provides robustness to transfer matrix implementations for large datasets. Once the recursion relations above are evaluated, the probability that base-pair *i* is covered by a ligand of type *s* is:

(7) \begin{equation*} p_s(i) = \frac{{\boldsymbol{w}}^L(i-1) \cdot {\boldsymbol{O}}_s \cdot {\boldsymbol{w}}^R(i) }{{\boldsymbol{w}}^L(0) \cdot {\boldsymbol{w}}^R(1)} \ \prod _{j=1}^{i-1}{\frac{n^L(i)}{n^R(i)}} \end{equation*}

The fact that Equation (7) above is equivalent to Equation (3) in the previous subsection follows by inserting Equation (6) in Equations (3), (4) and (5). Evaluating *p*_*s*_(*i*) for all base pairs, using Equation (7), again, requires *N* matrix multiplications. The total computational time of the method above, therefore, scales linearly with *N*. The utilization of the numerical procedure above, rather than direct application of Equations (3), (4) and (5), is crucial for long (typically *N* > 1000 base pairs) DNA molecules. Our Java implementation of the new scheme has been shown to work well for experimental barcode calculations where *N* ∼ 10^6^. For even longer molecules, we expect the method to be limited only by the computer internal memory storage capacity.

**Figure 4.**
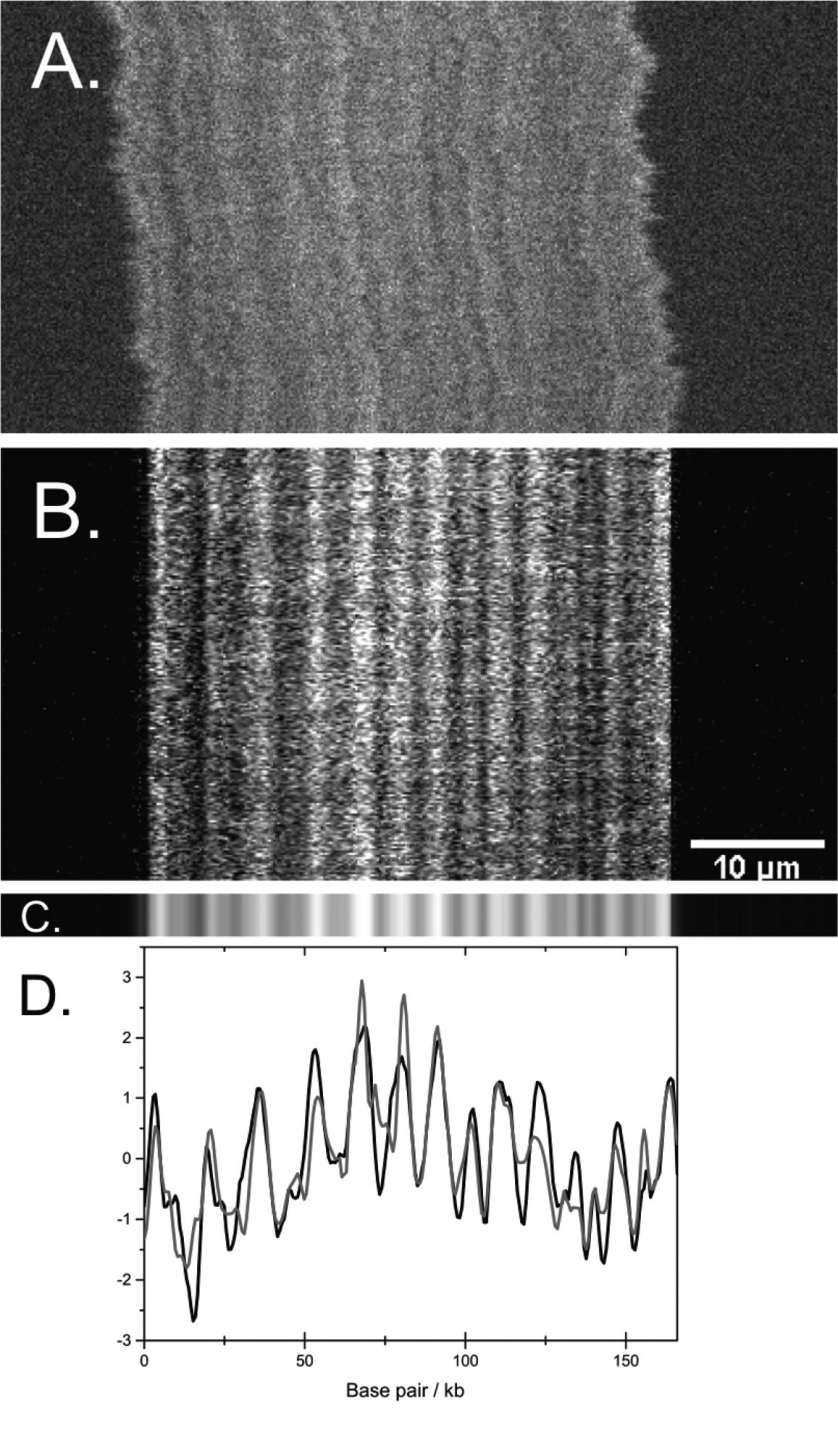
(**A**) Experimental raw kymograph for T4 DNA at 1:150 YOYO:netropsin in 0.05× TBE. Fluorescent images of DNA molecules were recorded at different times (time along the vertical axis). The sample was mixed in 5× TBE and diluted. (**B**) Aligned kymograph. (**C**) DNA barcode consisting of 20 lines generated from the average of the experimental kymograph. (**D**) Comparing the experimental (black) and theoretical (gray) barcodes.

Theoretical calculations require DNA sequences and the following input parameters, see Figure 3 and Equations (2)–(7) in the Supplementary Information: the concentrations, *c*_*s*_, for all ligands, sequence-specific binding constants, *K*_*s*_ and intra- and inter-ligand cooperativity parameters, σ_*s*, *s*_. Netropsin (here defined to be a type 1 ligand) is a minor groove binder, which has a strong preference for AT-quadromers. In all subsequent calculations, we set the netropsin binding constant to *K*_1_ = 5 · 10^5^ M^−1^ for quadromers containing one or several G's and C's. For quadromers containing A's and T's only, we used *K*_1_ = 10^8^ M^−1^ (18). For the fluorescent dye, YOYO-1 (here defined as a type 2 ligand), we used *K*_2_ = 10^10^ M^−1^ (32). For simplicity, we did not include any cooperativity, i.e. we set σ_1, 1_ = σ_1, 2_ = σ_2, 1_ = σ_2, 2_ = 1 in all calculations. As YOYO-1 is fluorescent, whereas netropsin is not, we set *s* = 2 in Equation (7). All sequences used were downloaded from the NCBI GenBank. In particular, the T4GT7 sequence was obtained by deleting a 3256 bp segment positioned between sites 165 255 and 168 510 in the T4 sequence. Binding probabilities for all theoretical sequences were then calculated, as described above. An illustrative example of the results of the transfer matrix approach for T4-DNA is found in Figure 4 (bottom), where an additional ‘blurring’ procedure has been performed (see subsequent sections), in order to mimic the limited experimental optical microscope resolution. Details of the experimental procedure and kymograph alignment procedures are provided in the next section.

### Comparing theory and experiments

In this section, we describe the procedure for comparing raw experimental data and theoretical predictions. A fit is characterized by two parameters: a best cross-correlation, $\hat{C}$, that describes how visually similar the barcode patterns are and a *P*-value that probabilistically describes how good the match is, compared to what would be expected ‘by chance’, when matching a small DNA fragment to a long theoretical barcode.

After appropriate alignment and molecule identification steps (see Supplementary Information), experimental data come in the form of a finite resolution barcode (due to the limiting optical microscope resolution) obtained at pixel level. Furthermore, the DNA molecules are not fully extended in the channel. In contrast, the theoretical barcode has a resolution down to single base pairs along the contour of the DNA molecule. To account for these differences, the quantity *i*, see Equation (7), which labels different base pairs, is first translated into length (μm) using a conversion factor, *l*, i.e. we replace *i* → *i*/*l*. We make the estimate, using the extension of a DNA with known contour length as reference, *l* = *l*_est_ = 4500 bp/μm for the T4 experiments (channel size = 100 × 150 nm^2^) and *l*_est_ = 3400 bp/μm (channel size = 100 × 100 nm^2^) for the experiments using the *E. coli* DNA fragments. To account for the difference in resolution between theory and experiment, a point-spread function in the shape of a Gaussian is convoluted with the theoretical barcode to simulate experimental conditions. In practice, the convolution is performed in Fourier space:

(8) \begin{equation*} p_{\rm theory}(i)= \textrm {ifft(fft(}p_s(i)\textrm {)}\cdot \textrm {fft(} \frac{1}{\sqrt{2\pi \cdot \sigma _{bp}^2}}e^{-\frac{(N/2 - i)^2}{2\sigma _{bp}^2 }}\textrm {))} \end{equation*}

where fft stands for ‘Fast Fourier Transform’, ifft is the inverse of fft and *N* is the number of base pairs in the barcode as before (we replace *N* → *N*/*l*, see above). We use the fft and ifft methods from the toolbox JTransform 2.4 (https://sites.google.com/site/piotrwendykier/software/jtransforms). The standard deviation of the point-spread function used is 0.3 μm, as determined by measuring the footprint of a single fluorescent quantum dot (16). Finally, we translate the different points along the barcode to pixels, utilizing yet another conversion factor, *f*, i.e. we make the further replacement *i* → *i*/*f*. We set *f* = 0.16 μm/pixel (a CCD camera with a pixel size of 16 × 16 μm^2^ and a 100× objective is used in the experiments). The above procedure for scaling the ‘horizontal’ axis of the theoretical barcode is only approximate, as *l* and *f* are not known exactly.

After the above procedure for approximate scaling of the barcode's horizontal axis, the quantitative comparison of experiments to theoretical barcodes is now a three-step procedure, requiring two fitting parameters: the imaging scale (number of base pairs per pixel, i.e. *lf*) and the position of the fragment along the theoretical barcode. The first step is to fine-tune the imaging scale. As *l* and *f* are not exactly known, we must allow the values of these parameters to vary slightly. In practice, we keep *f* fixed to the value given above and allow only *l* to vary between a minimum *l*_min_ and maximum *l*_max_ value centered on the estimate above. We use *l*_min_ = *l*_est_ − Δ and *l*_max_ = *l*_est_ + Δ with Δ =935 bp/μm. Secondly, we rescale both the theoretical and experimental barcodes’ ‘vertical’ axis such that the mean of the curve is zero and the standard deviation is one, i.e. (30)

(9) \begin{equation*} \delta P(i) = \frac{P(i) - \langle P(i)\rangle }{(\langle (P(i) - \langle P(i)\rangle )^2\rangle )^{1/2}} \end{equation*}

wherein *P*(*i*) is either the experimental signal, *I*_exp_(*i*), or *p*_theory_(*i*) with the rescaling of *i* to pixel levels as described above. The gray scales of the two barcodes are now comparable. The third, and final, step is to slide the experimental barcode across the theoretical barcode and compare them, using a cross-correlation measure:

(10) \begin{equation*} \chi (i_{\rm start},l) = \frac{1}{J} \sum _{i=1}^J \delta P_{\rm exp}(i) \cdot \delta P_{\rm theory }(i+i_{\rm start}-1, l) \end{equation*}

with *i*_start_ = 1, .., *I*, where *I* is the number of attempted placements of the experimental barcode onto the theoretical barcode and *J* is the number of pixels in the experiment. Since an experiment is performed on a DNA molecule with unknown direction, the experimental barcode is then flipped and the procedure above is repeated. For an experiment placed with parts ‘outside’ the end of δ*P*_theory_(*i*, *l*), we impose circular symmetry of the molecules studied here.

The best agreement between experiment and theory is decided by maximizing the cross-correlation, Equation (10), thereby providing us with the best start location, $\hat{i}_{\rm start}$, and best conversion factor, $\hat{l}$. The full set of cross-correlation values for the best conversion factor is:

(11) \begin{equation*} C(i_{\rm start})=\chi (i_{\rm start},\hat{l}) \end{equation*}

and the best value of the *C*(*i*_start_)'s is denoted by $\hat{C}$, i.e. $\hat{C}={\rm max}\lbrace C(1),...,C(I)\rbrace =\chi (\hat{i}_{\rm start},\hat{l})$. As demonstrated in the Results section, ‘large’ $\hat{C}$-values correspond to situations in which the agreement between theory and experiments is visually appealing.

In the Supplementary Information, we introduce a *P*-value for further quantifying the quality of match between experiments and theory (Equation (11) in the Supplementary Information). The reason for this is that direct use of $\hat{C}$ as a measure of agreement between experiment and theory can be problematic; note that we typically need to position a smaller experimental barcode along a much longer theoretical barcode. For a sufficiently small experimental segment, with few distinct features, the probability of getting a visually good agreement ‘by chance’ somewhere along the long barcode is high. Furthermore, since the quantity $\hat{C}$ is the largest (the ‘record’) out of *I* numbers, the larger *I* is (the longer the DNA sequence), the larger the ‘record’ $\hat{C}$ will be, in general. Therefore, $\hat{C}$ does not allow useful comparison for matching a DNA fragment of a given length onto short and long theoretical barcodes, respectively. For these reasons, we introduce a probabilistic approach by following the philosophy of (33), wherein a ‘null model’, corresponding to randomized theoretical barcodes, is introduced as a reference. Our approach adapts the approach in (33) to include *correlated* random numbers and *finite* experimental barcodes (*I* needs not be very large, as in (33)) and provide a *P*-value (Equation (11) in the Supplementary Information), i.e. the probability that a fit of the experimental barcode to a set of random barcodes is better than the best fit to the theoretical barcode $P{\rm -value} = \int _{\hat{C}}^\infty \phi (\hat{C}^{\prime }) d\hat{C}^{\prime }$ where $\phi (\hat{C})$ is the distribution for the best fit of the experiment on a set of random barcodes, constrained to be of the same length and of the same base-pair composition as the original sequence. The fact that the *C*(*i*_start_)-values in Equation (11) typically are correlated follows from the fact that when moving the experiment one pixel forward, *i*_start_ → *i*_start_ + 1, the theoretical profile may not have changed much. The full details of our approach is found in the Supplementary Information.

## RESULTS AND DISCUSSION

### Optimizing experimental conditions

The initial experimental focus of this paper is to optimize the DNA barcodes. There are two important factors to be considered: barcode information content and barcode reproducibility. We will discuss this in terms of two parameters; the SBR, defined in Equation (1), reflects how much YOYO that is bound to each DNA molecule and the IS, as quantified by Equation (2), reflects the number of distinct features (peaks and valleys) in the barcode, with respect to the background noise.

**Figure 5.**
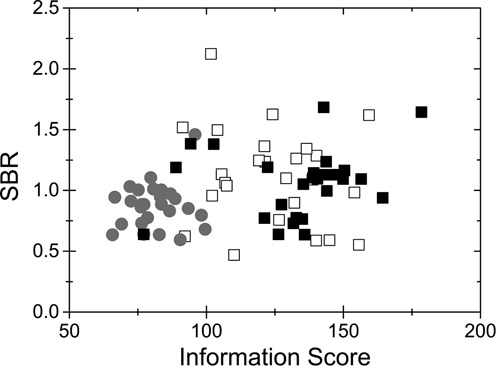
Comparing the SBR and IS under three experimental conditions. Gray circles: 0.5× TBE, open squares: 0.05× TBE and full squares: mixed at 5× TBE and diluted to 0.05× TBE. SBR and IS are defined in the Materials and Methods section.

Firstly, the IS for each barcode should be as high as possible. The degree of stretching of nanoconfined DNA increases with decreasing ionic strength (34), which increases the potential resolution of the barcode, in terms of base-pairs/pixel and therefore increases IS. In our proof-of-principle study, all experiments were conducted in 0.5× TBE buffer (16). Figure 5 shows SBR plotted as a function of IS for T4-DNA in 0.5× and 0.05× TBE. A vast majority of the molecules have a significantly larger IS at the lower ionic strength when the molecules are more stretched out. However, there is also a much larger spread in both SBR and IS at the lower ionic strength.

Secondly, all barcodes obtained from DNA fragments with identical sequence should be as similar as possible. An evenly stained sample is of crucial importance when single, unique fragments are considered, such as those from *E. coli* below. In a recent paper (29) the equilibration of YOYO on DNA was observed to be much faster at high ionic strengths, due to decreased electrostatic interactions between the dye and the DNA. The optimal conditions for high IS (low ionic strength) and reproducibility (high ionic strength) are, thus, orthogonal.

To satisfy both requirements, high IS and barcode reproducibility, we mix the samples at high ionic strength (5× TBE), to equilibrate the sample rapidly, and subsequently dilute the mixed sample to a low ionic strength (0.05× TBE), to maximize the stretching of the DNA within the nanochannels. This procedure yields molecules with a much larger information content than at 0.5× TBE, although also with a much smaller spread than for the sample mixed at 0.05× TBE (Figure 5). Mixing at high ionic strength and subsequent dilution is the protocol used for the study on DNA extracted from *E. coli* below.

### Experiments and theory for *E. coli* strain CCUG 10979

**Figure 6.**
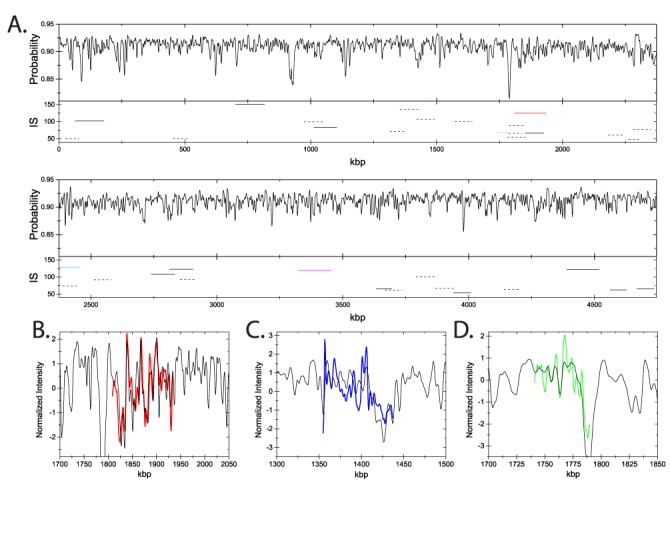
(**A**) The theoretical probability *p*_theory_(*i*) for YOYO binding to the full genome of *E. coli* strain CCUG 10979, calculated using the transfer matrix approach discussed in the Materials and Methods section. Horizontal lines represent the location of the best fits of 36 experimental *E. coli* fragments; the associated IS values are also displayed. Solid horizontal lines correspond to a *P*-value below 10% and dashed lines have a *P*-value above 10%. The five colored horizontal lines correspond to traces which are detailed in panels B–D, see also Figure 8. The best fit (colored curves) of three experimental fragments matched to the theoretical trace (black curves): (**B**) a representative fragment with a large best cross-correlation $\hat{C}$$\hat{C}$ value (0.771) and a small *P*-value (0.09 %); (**C**) a representative fragment with a small $\hat{C}$$\hat{C}$ (0.670) and a large *P*-value (37.1%); (**D**) a representative fragment with a large $\hat{C}$$\hat{C}$ (0.877) and a large *P*-value (33.3%). The colors of the fits correspond to the colors of the horizontal lines in (A).

One potential application of optical mapping is the characterization and identification of bacterial species and strains. We extracted DNA from the *E. coli* strain CCUG 10979 (= ATCC 8739), using conventional methods (see the Materials and Methods section). During the extraction protocol the DNA is fragmented, and barcodes for 36 such DNA fragments, with lengths ranging from 51.7 kb to 153.4 kb, were matched to the theoretical barcode of CCUG 10979, derived from the genome sequence (RefSeq, Acc. No. NC_010468.1). Figure 6A shows the full theoretical barcode of CCUG 10979 calculated using the transfer matrix approach (see the Materials and Methods section for details and input parameters). Figure 6A also shows the location of the best fits and the associated IS for each of the 36 fragments along the genome.

Figure 6B–D illustrates three common scenarios when matching experiments to a longer theoretical barcode. (B) The barcode match is ‘visually’ appealing (typically, large $\hat{C}$-value) and the match is also better than that obtained by matching to a random barcode (small *P*-value, see the Materials and Methods section). (C) The match is visually not good (small $\hat{C}$), and the match is as bad as when fitting to a random barcode. (D) The match is visually satisfactory, although the match to a random barcode is of equal quality. Note that there is no direct correlation between *p* and $\hat{C}$ (see Figure S7 in the Supplementary Information ) and, in general, that $\hat{C}$ cannot be used for reliably quantifying an experimental - theory match (see the discussion in the Materials and Methods section).

**Figure 7.**
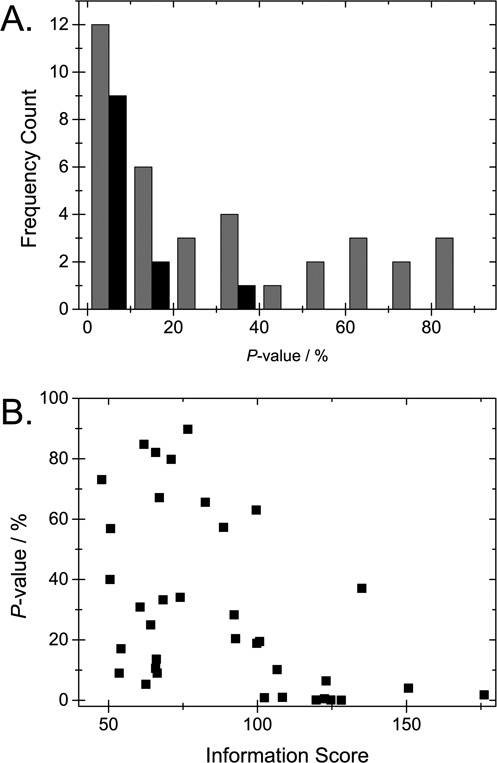
(**A**) Histogram (gray) of *P*-values obtained when fitting 36 experimental fragments from the *E. coli* strain CCUG 10979 to its theoretical barcode, and the 12 fragments with an IS above 100 (black). (**B**) Plot of the IS versus the *P*-value for the 36 fragments.

We henceforth use *P*-values to quantify agreement between experiments and theory and use the phrase ‘reliable match’, for scenarios when the experiment-theory match is significantly better than what we would expect by chance, i.e. when *P* < *P*_threshold_. Using *P*_threshold_ = 10% we find that 12 of the 36 fragments have a *P*-value that is below the threshold, although there is also a significant fraction with larger *P*'s (see Figure 7A). By investigating the horizontal bars in Figure 6A, where IS and *P*-values are indicated, we note that fragments with *P*-values larger than 10% are typically short and have a small IS.

Based on the findings above, we investigate whether assessing IS is a reliable way of excluding molecules with high *P*-values. The IS value is related to the number of distinct features (peaks and valleys) in the experimental trace (see the Materials and Methods section). Indeed, by analyzing the 36 DNA molecules, we observe that fragments with small *P*-values generally also have high IS (Figure 7B); barcodes with many distinct peaks and valleys (high IS) are more likely to show a good match to the correct position, compared to matches to random barcodes. To be able to identify fragments as belonging to a certain bacterial strain it will therefore be useful to use a ‘cut-off’ with a pre-determined IS. In the present study, we set this IS cut-off at 100. Doing so we obtain a group of fragments wherein a majority (75%) have a *P*-value below 10% (Figures 7A and B). An alternative approach for excluding molecules with a high *P*-value is to use a cut-off with respect to lengths of fragments. There is a clear, almost linear, correlation between the length of the fragment and the IS (see Figure S8 in the Supplementary Information) since longer fragments, on average, contain a larger number of distinct features.

The fact that three fragments with a high IS still have a *P*-value that is above 10% could potentially be explained by the fact that bacteria can spontaneously rearrange their genome. Since we study single DNA fragments we are sensitive to the fact that a small fraction of the bacteria contain a genome with rearrangements compared to the reference (35). The sensitivity to changes in the genome of a small fraction of bacteria in a population is a potential application of our optical mapping method in the future where such phenomena can be studied in detail.

### Comparing two closely related *E. coli* strains

**Figure 8.**
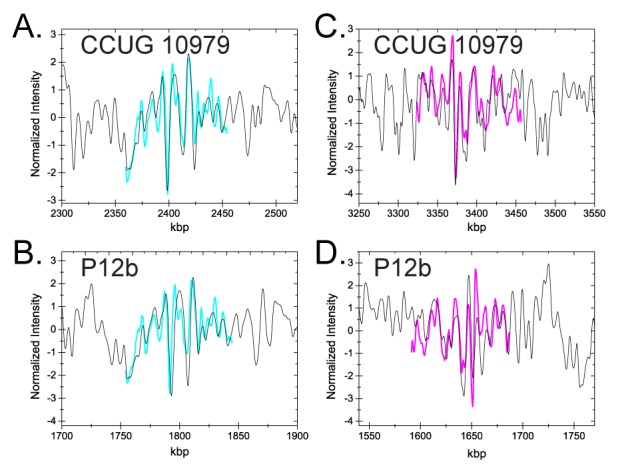
Fragments fitted to the theoretical genomes of CCUG 10979 and P12b, respectively. (A and B) A fragment with a good fit to both the correct strain CCUG 10979 and the P12b strain. (**A**) Location of one fragment (cyan curve) on the genome of the correct strain CCUG 10979 (black curve) with a *P*-value of 0.04% and a best cross correlation value of $\hat{C}=0.876$$\hat{C}=0.876$. (**B**) The same fragment as in (A) (cyan) located on strain P12b (black) with a *P*-value of 0.12% and $\hat{C}=0.848$$\hat{C}=0.848$. (C and D) A fragment with a good fit to CCUG 10979 and a bad fit to P12b. (**C**) Location of one fragment (magenta) on the genome of the correct strain CCUG 10979 (black curve) with a *P*-value of 0.13% and $\hat{C}=0.732$$\hat{C}=0.732$. (**D**) The same fragment as in (C) (magenta) located on strain P12b (black) with a *P*-value of 23% and $\hat{C}=0.6231$$\hat{C}=0.6231$.

The CB assay could in the future potentially be used in clinical settings to identify bacterial infections. To explore the feasibility of using the assay for the identification of bacterial isolates, we generated the full theoretical barcode for the *E. coli* strain P12b. The genome of the *E. coli* strain P12b has been sequenced (Acc. No. NC_017663); strain P12b is the fully genome sequenced *E. coli* strain that is most closely related to CCUG 10979 according to a NCBI Genomic BLAST dendrogram comparing complete genome sequences of *E. coli* strains (http://www.ncbi.nlm.nih.gov/genome/167, Table 1). Since the strains are closely related, we expect some fragments to fit well to both strains. In Figure 8 we give representative examples of a fragment of DNA from CCUG 10979 that fits well to both the correct strain and strain P12b (A and B) and a DNA fragment that fits well only to strain CCUG 10979 (C and D). Note that, even though the fragment in Figure 8A and B has a very low *P*-value for both strains, the fragment is located at different positions along the respective genomes.

**Table 1.** Genome sequenced *E. coli* strains compared to ATCC 8739 (CCUG 10979), Acc. No. NC_010468.1 using BLAST

*E. coli* strain	Total score (BLAST)	Accession number
P12b	1.051E7	NC_017663.1
HS	9.524E6	NC_009800.1
str. K-12 substr. MG 1655	9.495E6	NC_000913.3
O 104:H4 str. 2011C-3493	9.013E6	NC_018658.1
O 157:H7 str. Sakai	8.559E6	NC_002695.1
UMN 026	8.374E6	NC_011751.1
IAI 39	7.985E6	NC_011750.1
O83:H1 str. NRG 857C	7.771E6	NC_017634.1
The total score is the sum of scores of all aligned sequences. The higher the score the higher the similarity between the aligned sequences.		

**Figure 9.**
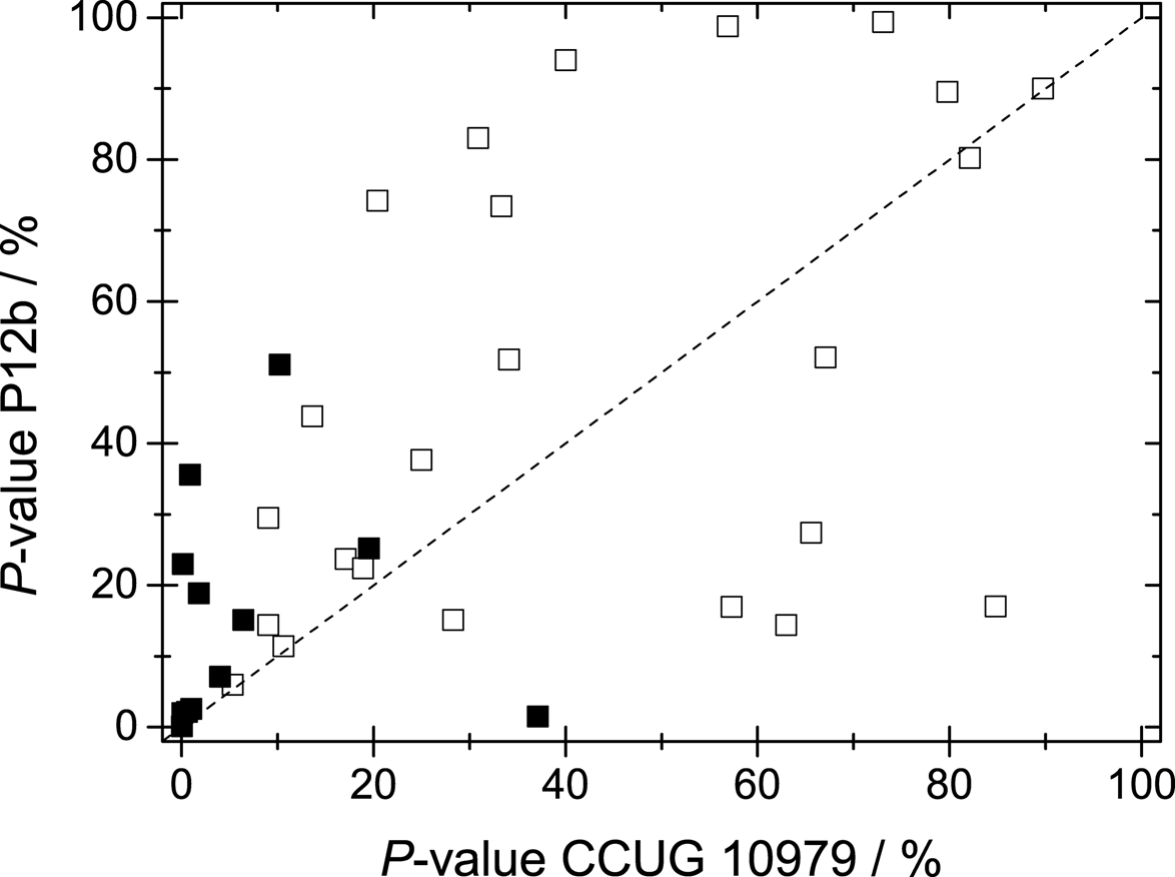
*P*-value for all 36 fragments (squares) fitted to the correct strain CCUG 10979 (x-axis) and the reference strain P12b (y-axis). The 12 fragments with an IS above 100 are shown as full symbols. The dashed line corresponds to equal values for both strains.

Figure 9 shows the *P*-values for all 36 fragments matched to the barcodes of strains CCUG 10979 and P12b. While there is a slight trend that the *P*-values are lower for the correct strain, the resolution in separating the two is poor. However, when using the IS threshold introduced above, a vast majority of the DNA fragments have a significantly lower *P*-value than for strain P12b; there is only one significant false positive.

### Identification of *E. coli* strains from a small database

**Figure 10.**
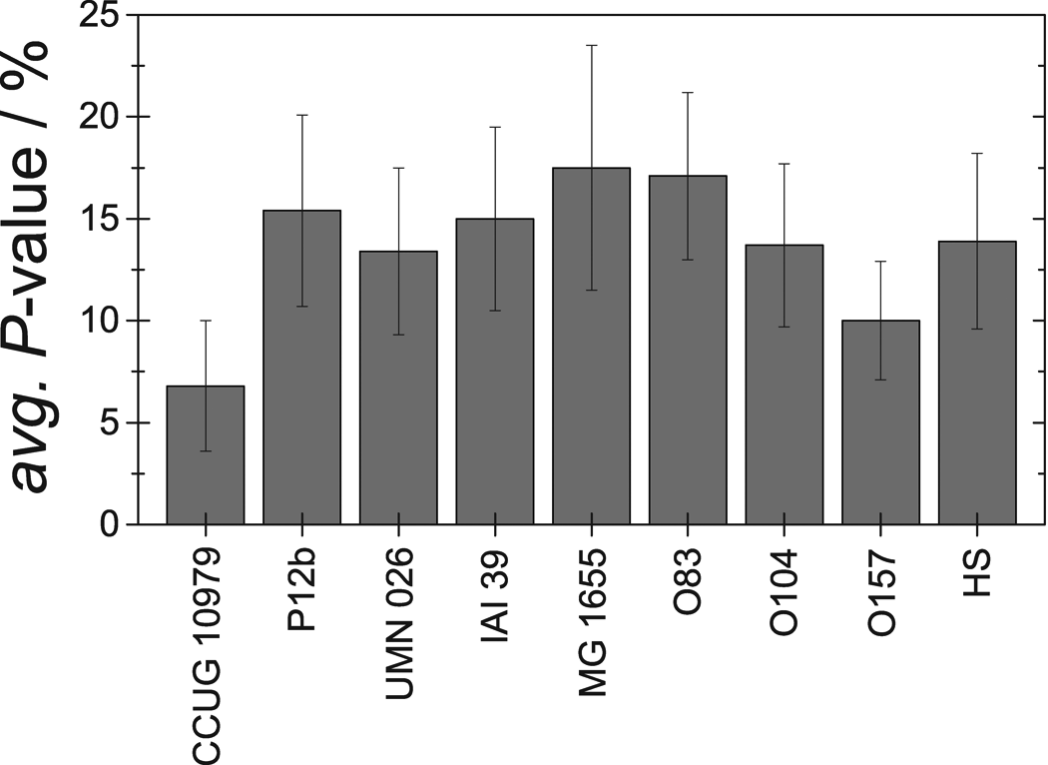
Average *P*-value and standard error for the correct strain (CCUG 10979) as well as the eight reference strains for the 12 fragments with an IS above 100.

**Figure 11.**
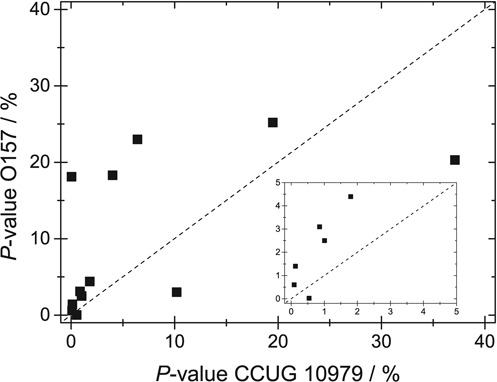
*P*-value for the 12 fragments with an IS above 100 when fitted to the correct strain CCUG 10979 (x-axis) and the reference strain O157 (y-axis). The dashed line corresponds to equal values for both strains. The inset shows a zoom in of the data on the low *P*-value regime.

As a proof-of-principle, we demonstrate that theoretical barcodes from different *E. coli* strains are sufficiently different so that the CB assay can differentiate them. To that end, we determined *P*-values for the 12 DNA fragments from strain CCUG 10979 with IS values larger than 100, matched to the theoretical barcodes for nine different *E. coli* strains. The strains are listed by NCBI as reference genome sequenced *E. coli* strains. A comparison of CCUG 10979 and these eight strains was performed and the resulting total BLAST scores are shown in Table 1. The total score is the sum of scores of all aligned sequences. Figure 10 shows that the average *P*-value is significantly lower for DNA fragments from strain CCUG 10979 than for any other strain. Four strains are statistically well separated from the correct one, three are on the very limit to be statistically resolved while strain O157:H7 Sakai is hardest to exclude. However, using the average of all *P*-values could give a false picture, since it will be strongly affected by a single ‘outlier’ that increases the average dramatically. Furthermore, fragments that have a high *P*-value for both strains compared should not be taken into account at all when comparing them. More information can potentially be obtained by, as in Figure 9, instead comparing the *P*-values for individual fragments when fitted to two strains. Figure 11 shows such a comparison for CCUG 10979 and O157:H7 Sakai (similar plots for all strains can be found in Figure S9 in the Supplementary Information). A vast majority of the fragments fit better to strain CCUG 10979. The major ‘false positive’ has a high *P*-value also for strain O157:H7 Sakai (∼20%) and should, therefore, not be taken into account when comparing the two strains. Rather, when comparing fragments that have a *P*-value lower than 5% for at least one of the strains, only 2 out of 9 fragments fit better to strain O157:H7 Sakai. The assay used is thus able to resolve these two strains, provided the IS cut-off and *P*-value tools are used.

## CONCLUSION AND OUTLOOK

We introduce the theory and experimental results for an optical mapping method for single DNA molecules, based on Competitive Binding (CB) of YOYO and netropsin and stretching in nanochannels. The assay produces emission intensity variations along nanoconfined DNA molecules, a barcode, that reflects the underlying sequence with kb resolution. To relate the resulting barcodes to the underlying DNA sequence, we extend existing theories, based on a transfer matrix approach, to ultra-long DNA pieces and an arbitrary number of competing ligand types. The multi-ligand transfer matrix method introduced here is a convenient technique for calculating theoretical barcodes.

Using the experimentally obtained barcodes and the theoretical framework, we demonstrate that it is possible to identify a specific *E. coli* strain (CCUG 10979) from a reference database of genome sequences of nine *E. coli* strains. Our identification protocol utilizes a *P*-value for an experiment-theory match and an IS threshold. IS can be efficiently calculated and is a powerful method for discarding molecules with a large *P*-value. The method should find applications to scenarios beyond the present study, for instance, for screening clinical isolates of an infectious outbreak.

Since IS is closely related to the length of each filament, we foresee that an even faster and more efficient bacterial identification could be done by developing techniques to extract longer DNA pieces from bacteria. Our results suggest that we do not need exceptionally long fragments to get a unique identification but, rather, only a small increase in the size of the fragments extracted will improve the resolution of the assay significantly.

## SUPPLEMENTARY DATA

Supplementary Data are available at NAR Online.

SUPPLEMENTARY DATA
