# Supplementary material for: Competitive binding-based optical DNA mapping for fast identification of bacteria - multi-ligand transfer matrix theory and experimental applications on Escherichia coli
Source: Nucleic Acids Res. 2014 Jul 10;42(15):e118. doi: 10.1093/nar/gku556 (PMC4150756; doi:10.1093/nar/gku556)
Supplement: SUPPLEMENTARY DATA [file supp_42_15_e118__index.html]

Competitive binding-based optical DNA mapping for fast identification of bacteria - multi-ligand transfer matrix theory and experimental applications on Escherichia coli — SUPPLEMENTARY DATA 

# Competitive binding-based optical DNA mapping for fast identification of bacteria - multi-ligand transfer matrix theory and experimental applications on *Escherichia coli*

## SUPPLEMENTARY DATA

**Files in this Data Supplement:**

- Supplementary Data
