# Supplementary material for: Competitive binding-based optical DNA mapping for fast identification of bacteria - multi-ligand transfer matrix theory and experimental applications on Escherichia coli
Source: Nucleic Acids Res. 2014 Jul 10;42(15):e118. doi: 10.1093/nar/gku556 (PMC4150756; doi:10.1093/nar/gku556)
Supplement: SUPPLEMENTARY DATA [file supp_gku556_nar-00904-met-n-2014-File001.pdf]

Published online – *Nucleic Acids Research, 2014, Vol. –, No. –* **1–5**
doi:10.1093/nar/gkn000

Supplementary: Competitive binding-based optical DNA mapping for fast identification of bacteria – multi-ligand transfer matrix theory and experimental applications on *Escherichia coli*

Adam N. Nilsson [1], Gustav Emilsson [2], Lena Nyberg [2], Charleston Noble [1,5], Liselott Svensson Stadler [3], Joachim Fritzsche [4], Edward R. B. Moore [3], Jonas O. Tegenfeldt [5], Tobias Ambjörnsson [1], Fredrik Westerlund [2].*

[1]Department of Astronomy and Theoretical Physics, Lund University, Sölvegatan 14A, 223 62 Lund, Sweden and [2]Division of Chemistry and Biochemistry, Department of Chemical and Biological Engineering, Chalmers University of Technology, Kemivägen 10, 412 96 Göteborg Sweden, Sweden and [3]Department of Infectious Diseases, Göteborg University, Guldhedsgatan 10, 413 46 Göteborg, Sweden and [4]Department of Applied Physics, Chalmers University of Technology, Kemivägen 10, 412 96 Göteborg Sweden, Sweden [5]Division of Solid State Physics, Department of Physics, Lund University, PO 118, 221 00 Lund, Sweden

Details of the kymograph alignment and DNA identification procedures

In experiments fluorescent images of extended, netropsin-YOYO stained, DNA molecules are recorded sequentially, see Figure S1 (top) for an example of a resulting raw kymograph. There are two challenges for extracting a barcode from such raw data which can subsequently be compared to theoretical predictions: First, the data must be aligned, *i.e.*, center-of-mass fluctuations and local conformational fluctuations seen in Figure S1 (top) must be corrected for. The experimental data can be time-averaged only after this alignment has been performed. Second, the time-averaged signal contains both a background region (the molecule does not occupy the full field of view) and a region containing the DNA molecule. The latter region must be identified, before comparison to theory can be made.

Concerning the first step, *i.e.* aligning experimental data so that time-averages can be performed, we first apply a moving average to the experimental data with a window size of 5 pixels. This step is performed only for alignment purposes, in order to avoid aligning noise to noise. The final time-averaged barcode is presented without the moving average. After this step, the experimental data can be aligned using the local box-stretching approach presented in (1). However, in order to reduce computational efforts associated with the global minimization procedure of the method in (1), we start by performing a rough first alignment before the approach in (1) is applied. Our rough alignment procedure identifies the starting and finishing pixel of the region containing the DNA, at each time-frame, and aligns the end-points. Our method for identifying the DNA molecule's two ends proceeds by introducing a cumulative sum, $S(i)$ of the experimental signal, $input$, as follows:

$$\begin{aligned} mean &= \langle input\rangle - (\langle input\rangle \\ &\quad -\langle background\rangle)\cdot\alpha\cdot\frac{1}{1+e^{\frac{\langle input\rangle/\sigma-\beta}{\gamma}}} \\ S(0) &= input(0)-mean \\ S(i) &= S(i-1) \\ &\quad +input(i)-mean \end{aligned} \quad (1)$$

where σ is the standard deviation of the input signal and $\langle X\rangle$ denotes average of X. The end points of the DNA molecule are determined by the global maxima and minima of the cumulative sum, $S(i)$. To make the cumulative sum steeper, so that the global minimum and maximum are more pronounced, the mean is above multiplied with a Fermi-Dirac distribution. The following values were empirically found to be satisfactory for our data: $\gamma=0.1$, $\alpha=0.25$ and $\beta=5$. Once the start and end point of the DNA molecules are aligned, *i.e.* the extrema of $S(i)$ are determined, we apply the method in (1), see Figure S1 (middle) for a resulting aligned kymograph, and Figure S1 (bottom) for the associated time-averaged barcode.

The second step, before the time-averaged barcode can be compared to theoretical predictions, is to "cut out" the barcode's background. We do this in a simplistic way, by simply transforming the signal into a binary signal: if a value at a certain pixel is below the mean it gets the value zero and if the value is above the mean it gets the value one. We now start at the beginning of the binary signal and continues forward until we reach a pixel with the value one, this pixel represents the beginning of the DNA region. The same procedure is used to find the end pixel, but now we start from the end and move

*A.N. and G.E. contributed equally. T.A and F.W contributed equally. To whom correspondence should be addressed. Tel: +44 000 0000000; Fax: +44 000 0000000; Email: fredrik.westerlund@chalmers.se, tobias.ambjornsson@thep.lu.se

© 2014 The Author(s)
This is an Open Access article distributed under the terms of the Creative Commons Attribution Non-Commercial License (http://creativecommons.org/licenses/by-nc/2.0/uk/) which permits unrestricted non-commercial use, distribution, and reproduction in any medium, provided the original work is properly cited.

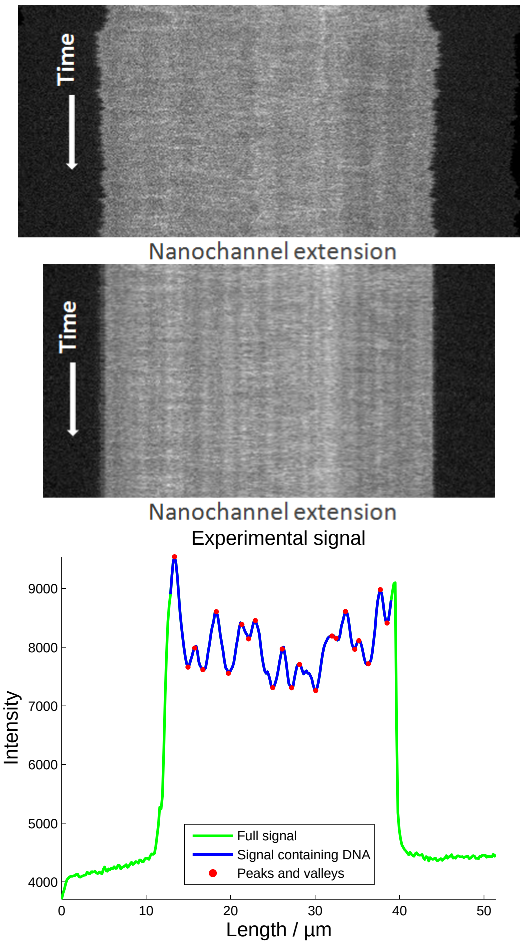

Figure S1. Kymograph alignment and DNA identification procedures applied to an experiment on a T4 bacteriophage: (top) experimental data before alignment, (middle) after alignment, and (bottom) time-averaged experimental signal. The green and the blue curve together makes up the entire experimental barcode. The blue curve corresponds to the region containing the DNA molecule, as identified using our procedure. The experiment is performed with a salt concentration of 0.05X TBE. Indicated as red dots are “robust” maxima and minima, which are used for calculating the information score (IS), see main text.

towards the beginning. This method gives a satisfying result, as can be seen in Figure S1 (bottom), where the green part of the barcode is the background and the blue part corresponds to the DNA region.

As a consistency check of the first step above, *i.e.*, the alignment procedure, we analyze noise levels around the time-averaged barcode. To that end, we subtract the time averaged barcode from the aligned kymograph in Figure S1, resulting in Figure S2 (top). The noise-level kymograph still has traces of the actual signal but these traces are of the same order as the noise. A histogram of the noise, see Figure S2 (bottom), reveals that the noise is Gaussian with mean zero.

Details of the multi-ligand transfer matrix method

In this section we provide further details on our multi-ligand transfer matrix method, introduced in the Methods section of the main text. In particular, we provide explicit expressions for the transfer matrix elements which allow straightforward

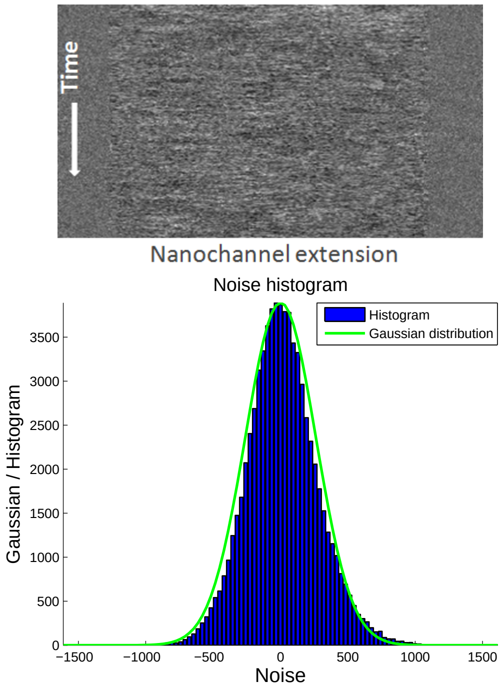

Figure S2. (Top) Noise levels around the aligned experimental kymograph in Figure S1. The time averaged signal in Figure S1 (bottom) is subtracted from the aligned kymograph in Figure S1 (middle). (Bottom) Histogram over the noise from the top plot. The green curve is a Gaussian distribution fitted to the histogram.

automated computation for arbitrary number of ligands of arbitrary size. We also provide illustrations of our choice of state enumeration scheme. A cartoon of the problem is found in figure S3 where the the different statistical weights needed for the transfer matrix approach are also given (see also main text).

The explicit form of the transfer matrices, $\boldsymbol{T}_i$ (see main text), in terms of the physical parameters relies on an particular enumeration scheme of the possible states for a given base-pair i. We choose to use m ($m=1,...,M$) as a label for the different states and employ the enumeration scheme in Figure S4, see also main text. There are, in total, $M=\sum_{\alpha=1}^{S}\lambda_\alpha+1$ number of states for each base-pair.

Let us now provide explicit forms for the transfer matrix elements which allows automated computation, see Figure 3 in the main text for illustrations. With the choice of enumeration scheme in Figure S4 in mind and using the statistical weights illustrated in Figure 2 in the main text we have:

- **A.** *Sites i and $i+1$ are both empty:*

$$T(i;\,1,1)=1. \tag{2}$$

Using the enumeration scheme in Figure S4, this case corresponds to sites i and $i+1$ both being in state 1,

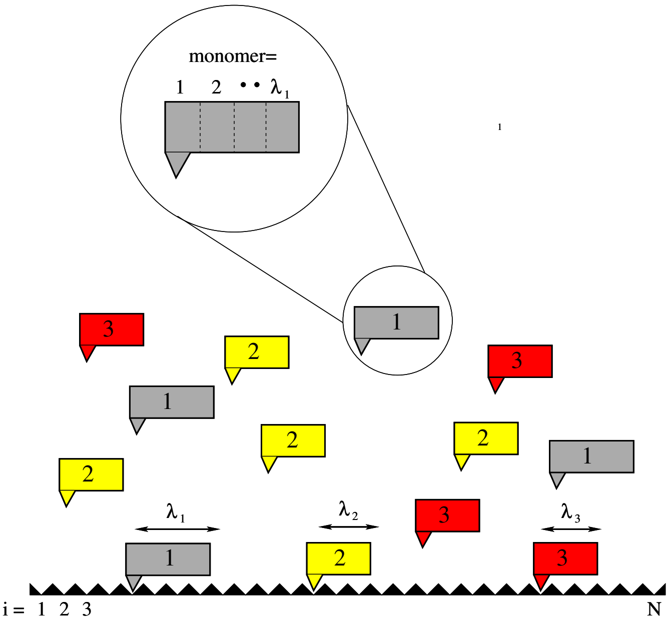

Figure S3. Cartoon of a model for competitive binding (CB) to DNA. S types of ligand species compete for binding to a one-dimensional lattice (DNA molecule), with N binding sites. When bound, a ligand of type s ($s=1,2,...,S$) covers λ_s sites and the ligands cannot bind to the same sites. The binding of ligand species, s, is characterized by its binding constants, $K_s(i)$, to different sites i ($i=1,...,N$). Inter- (and intra-) ligand interactions between species s and s' appear through the cooperativity parameters, $\sigma_{s,s'}$. We use $S=3$ in this cartoon, in Figure S4 and in the figures in the main text, for illustrative purposes, but the theoretical transfer matrix framework introduced in the main text (Methods) and here applies to an arbitrary number of ligand types.

- **B.** *Site i is empty and a ligand of type s has its first monomer at site $i+1$:*

$$T_i(i;1,1+\sum_{\alpha=1}^{s}\lambda_\alpha)=1, \qquad \forall s, \tag{3}$$

For instance, for $s=1$ (*i.e.*, "gray" ligands in Figure S4), the case above corresponds to site i being empty and and site $i+1$ is in state λ_1+1, see Figure S4.

- **C.** *Ligand of type s has its last monomer at site i and site $i+1$ is empty:*

$$T_i(i;\,2+\sum_{\alpha=1}^{s-1}\lambda_\alpha,1)=1, \qquad \forall s, \tag{4}$$

where, for $s=1$, it is to be remembered that the sum in the first argument is zero (since $\sum_{\alpha=1}^{0}=0$). For instance, for $s=1$ (*i.e.* "gray" ligands in Figure S4), the case above corresponds to site i being in state 2 and site $i+1$ is in state 1, see Figure S4.

- **D.** *Ligand of type s covers both site i and site $i+1$ (first monomer is not at site i):*

$$T(i;\,m+1,m)=1 \tag{5}$$

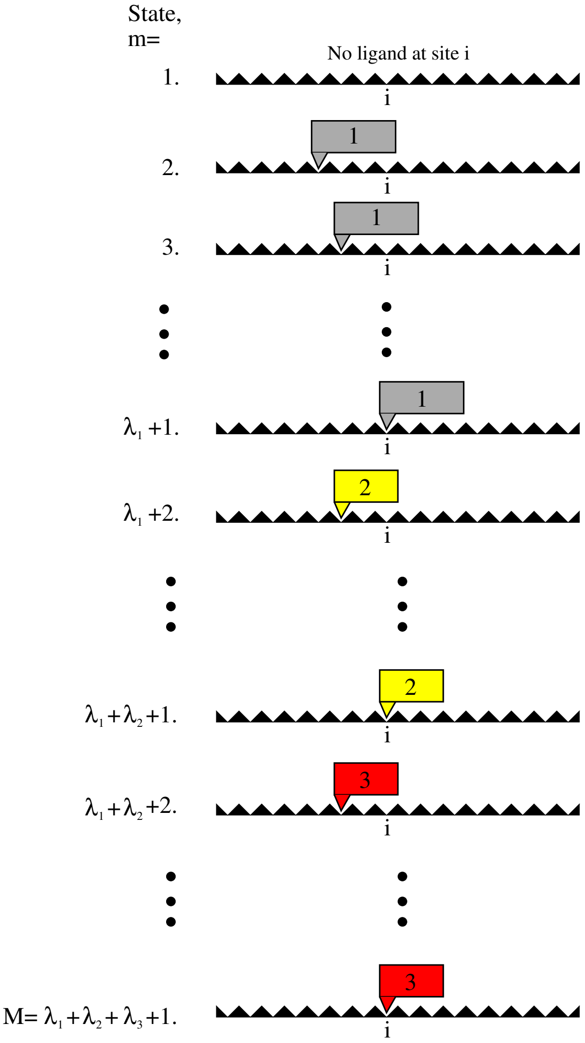

Figure S4. Enumeration of all possible states, labeled by m, of a base-pair i. In total, there are $M=\sum_{\alpha=1}^{S}\lambda_\alpha+1$ different states, where S is the number of ligand species. For illustrative purposes, we here display three types of ligands. However, the formalism introduced here applies to an arbitrary number of ligand species.

for all integers $m\in\{2+\sum_{\alpha=1}^{s-1}\lambda_\alpha,...,-1+\sum_{\alpha=1}^{s}\lambda_\alpha\}$ $\forall s$.

- **E.** *Ligand of type s at site i neighbors a ligand of type s' at site $i+1$:*

$$T(i;\,2+\sum_{\alpha=1}^{s-1}\lambda_\alpha,1+\sum_{\alpha=1}^{s'}\lambda_\alpha)=\sigma_{s,s'}, \qquad \forall s,s'. \tag{6}$$

For instance, if there is a "gray" ligand (type 1) at site i and a "yellow" ligand (type 2) at site $i+1$, then following the enumeration in Figure S4, site i is in state 2, whereas site $i+1$ is in state $\lambda_1+\lambda_2+1$.

- **F.** *Ligand of type s has its first monomer at site i*

$$T(i;\,1+\sum_{\alpha=1}^{s}\lambda_\alpha,\sum_{\alpha=1}^{s}\lambda_\alpha)=c_sK_s(i), \qquad \forall s. \tag{7}$$

An example of such a scenario is that a “gray” ligand binds with its first monomer to site i. Using the enumeration in Figure S4, this case corresponds to site i being in state λ_1+1 and site $i+1$ in state λ_1.

Matrix elements are calculated for all $s,s'=1,...,S$ above, and all remaining elements are equal to zero. The explicit transfer matrix elements given in equations (**2**)-(**7**) generalize the transfer matrices given in reference (2) (where $S=2$ was considered) to the case of site-specific competitive binding of S (≥ 2) species.

Details on the p-value approach

In our probabilistic approach for gauging the quality of match between experiments and theory, we follow the philosophy of (3), wherein a “null model”, corresponding to randomized theoretical barcodes, is introduced as a reference. Our approach, as detailed below, adapts the approach in (3) to include finite experimental barcodes and *correlated* random numbers – note that the $C(i_{\rm start})$-values in equation **(11)** in the main texttypically are correlated (see main text). As noted in the main text, our approach uses a p-value defined as:

$$p\text{−value} = \int_{\hat{C}}^{\infty} \phi(\hat{C}')d\hat{C}' \qquad (\mathbf{8})$$

where $\phi(\hat{C})$ is the distribution for the best fit of the experiment on a set of random barcodes, constrained to be of the same length and of the same base-pair composition as the original sequence, see Figure S5.

In order to obtain $\phi(\hat{C})$, and, hence, the p-value in Eq. (**8**), we use results from extreme value statistics (4) and proceed as follows: n random barcodes are created; we calculate and store *all* C-values (not just the best value), by comparing to the experimental barcode, see equation **(11)** in the main text. We then create a histogram of the C's and extract the sample mean μ and the standard deviation σ. We subsequently assume the distribution of C's to be Gaussian:

$$p_{\rm random}(C) = \frac{1}{\sqrt{2\pi\sigma^2}} \cdot \exp[-\frac{(C-\mu)^2}{2\sigma^2}]. \qquad (\mathbf{9})$$

As demonstrated in Figure S6, this assumption is satisfactory for our type of data (blue bars and black solid curve). To find the distribution for the “best” C, we use the fact that the probability density for the largest of I *independent*, identically distributed, random numbers is $\phi(\hat{C}) = Ip(\hat{C})[\int_{-\infty}^{\hat{C}} p(C)dC]^{I-1}$.(5) Also using equation (**9**) we find:

$$\phi(\hat{C}) = rI \cdot p_{\rm random}(\hat{C})[\frac{1}{2}(1+{\rm erf}(\frac{\hat{C}-\mu}{\sqrt{2\sigma^2}}))]^{rI-1}, \qquad (\mathbf{10})$$

where ${\rm erf}(x)$ is the error-function. Eq. (**10**) is displayed in Figures S5 and S6. In order to extend the standard result above to *correlated* data we simply replaced I by $I_{\rm eff}=rI$ above, with r being an effective inverse correlation length. The result above with $r=1$ is exact for independent (and hence uncorrelated) random numbers. Interestingly, we find empirically that the functional form given in equation (**10**) fits nicely also to our correlated data provided we choose $r \neq 1$, see Figure S6 (green bars and red solid curve). Thus, in practice, r is obtained by fitting to equation (**10**). To obtain an intuitive picture as to why we can simply use the functional form above also for correlated data, let us imagine a set of random numbers, I, where every consecutive set of five numbers are *perfectly* correlated; different sets are, however, independent. For this case, we can turn the I random numbers into a set of independent random numbers by keeping only $I/5$ independent numbers, and, thus, equation (**10**) applies with $r=1/5$. The value of r is found empirically to be rather insensitive to the choice of the length I. This insight might, in the future, allow us to extract r from a small set of random barcodes by fitting to equation (**10**). Such numerical speed up for p-value calculations will be of importance when handling large data sets and ultralong DNA molecules.

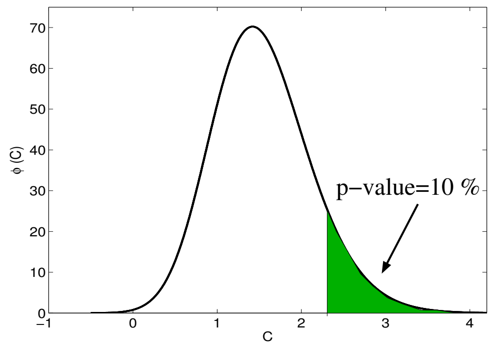

Figure S5. Illustration of the probability density, $\phi(\hat{C})$, for the largest value, $\hat{C}$, of N Gaussian random numbers, see equation (**10**). The green area corresponds to the p-value as defined in equation (**8**) and given explicitly, for Gaussian random numbers, in equation (**11**). We used parameters: $\mu=0$, $\sigma^2=1$ and $rI=10$.

Finally, we note that the p-value, equation (**8**), can be explicitly evaluated, using the fact that $\phi(\hat{C}) = (d/d\hat{C})[\int_{-\infty}^{\hat{C}} p(\hat{C}')d\hat{C}']^{rI}$. Therefore, in our case, we have

$$p\text{−value} = 1 - [\frac{1}{2}(1+{\rm erf}(\frac{\hat{C}-\mu}{\sqrt{2\sigma^2}}))]^{rI} \qquad (\mathbf{11})$$

where we used equation (**10**) and the fact that ${\rm erf}(\infty)=1$ and that ${\rm erf}(-x)=-{\rm erf}(x)$. Equation (**11**) provides an explicit expression for the p-value, for gauging the quality of match of an experimental barcode to a theoretical barcode of “length” I (more precisely, I, the number of attempts at placing the experiment on top of the theoretical barcode). The quantities r, μ and σ are estimated, using the method described above, again, see Figure S6 for an example of our procedure at work.

Finally, let us discuss some future challenges for the probabilistic approach for quantifying theory-experimental agreement introduced here. Firstly, note that our p-value is based on using random barcodes as reference. Future database tools may be sophisticated. For instance, rather than random DNA barcodes as reference, it could be advantageous

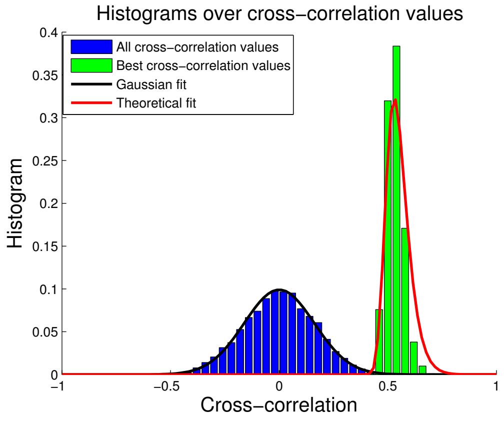

Figure S6. Determining the three fitting parameters (μ, σ and r) in the probability density for the best cross-correlation value for randomized barcodes, see equation **10**. The blue histogram contains all the cross-correlation values when comparing the experiment with a theoretical barcode using a randomized DNA sequence, where $I = 14562$ attempts were used. From this histogram a mean μ and a variance σ are extracted by fitting (black curve). We obtained $\mu = 2.9 \cdot 10^{-4}$ and $\sigma = 0.16$. The green histogram contains only the maximum cross-correlation values, $\hat{C}$, from these comparisons. The red curve is equation **10**, where fitting was used to extract the inverse correlation length r. Here, $r = 0.12$ was obtained.

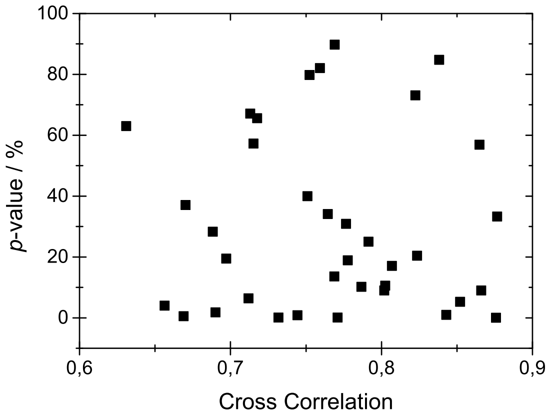

Figure S7. Cross-correlation ($\hat{C}$-value) and p-values for all 36 experimental DNA fragments fitted onto the genome of CCUG 10979. We see no direct correlation between a low $\hat{C}$-value and a low p-value. As discussed in the main text this can mainly be explained by the difference in the information content of the different barcodes.

to use the full set of known sequences in the database as a reference, and also include expected experimental molecule-to-molecule fluctuations. Secondly, we note that in some types of applications, one may be interested in studying the *uniqueness* of best placement of the experimental barcode on the theoretical counter part: are there several, well-separated (uncorrelated), positions along the theoretical barcode that provide an almost as good match as the best match? Addressing such an issue, along the probabilistic lines introduced here, would require not only the p-value for the best match but also a p-value for the second best match *et c*.

Supplementary Experimental Results

In the main text we discuss the relationship between a visually appealing fit (large $\hat{C}$) and a reliable experiment-theory match (low p-value). Figure S7 compares $\hat{C}$ and p-value for the 36 fragments studied. We see that there is no direct correlation between the two. This result is due to the fact that there are some, in general shorter, fragments that yield a visually "good" fit but where the probability of an equally good fit to a random sequence is as likely.

In the main text we introduce an Information Score (IS) that reflects how many distinct features, peaks and valleys, there are in each barcode. Figure S8 shows that IS is linearly related to the length of a fragment. This is expected since a longer fragment will on average have more distinct features than a short fragment and the increase should on average scale linearly with length.

In the main text we use p-values to separate the correct strain CCUG 10979 from eight other strains. In Figure S9 we compare p-value for CCUG 10979 and each of the other strains for individual fragments. We see that for fragments with IS above 100 a vast majority of the fragments have a lower p for the correct strain.

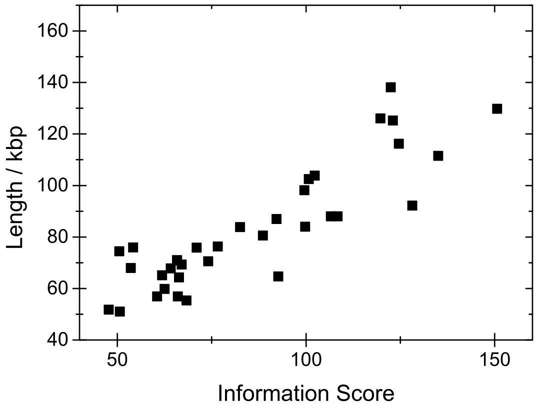

Figure S8. Fragment length as a function of Information Score (IS) for all 36 experimental DNA fragments (see main text). We observe a clear, almost linear, relation between the two. This is expected since a longer fragment on average will contain more values and peaks, which leads to a higher IS.

REFERENCES

1. Robert L. Welch and Robert Sladek and Ken Dewar and Walter W. Reisner. Denaturation mapping of saccharomyces cerevisiae. *Lab Chip*, 12:3314–3321, 2012.
2. V.B. Teif and K. Rippe. Calculating transcription factor binding maps for chromatin. *Briefings in Bioinformatics*, 13:187–201, 2011.
3. Karlin S and Altschul SF. Methods for assessing the statistical significance of molecular sequence features by using general scoring schemes, 1990.
4. Ang A H-S and Tang W H. *Probability Concepts in Engineering Planning and Design, Vol. 2: Decision, Risk, and Reliability*. John Wiley & Sons, 1984.
5. Schmittmann B and Zia RKP. weather records: Musings on cold days after a long hot indian summer. *American Journal of Physics*, 67:1269, 1999.

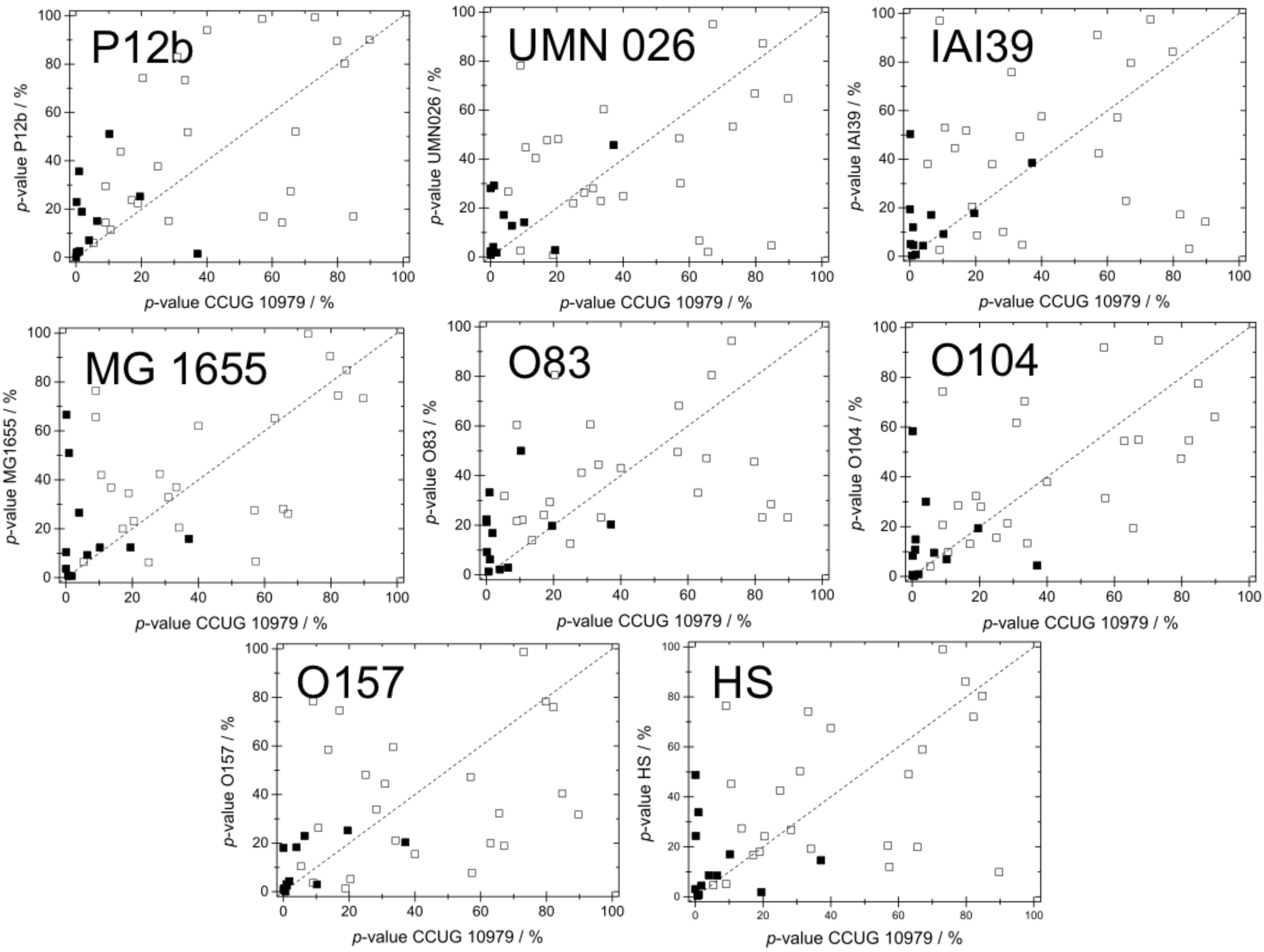

Figure S9. Individual graphs for comparing each reference strain to CCUG 10979. p-value for all 36 fragments (squares) fitted to CCUG 10979 (x-axis) and each of the reference strains (y-axis). The 12 fragments with an information score (IS) above 100 are shown as full symbols. The dashed line corresponds to equal values for both strains.
